# Pancytopenia due to iron deficiency worsened by iron infusion: a case report

**DOI:** 10.1186/1752-1947-1-175

**Published:** 2007-12-07

**Authors:** Apar Kishor Ganti, Nicole A Shonka, William D Haire

**Affiliations:** 1 Department of Internal Medicine, Division of Oncology/Hematology, University of Nebraska Medical Center, Omaha, NE, USA; 2 VA Medical Center, Omaha, NE, USA; 3 Department of Internal Medicine, University of Nebraska Medical Center, Omaha, NE, USA

## Abstract

**Introduction:**

Iron deficiency anemia is commonly associated with thrombocytosis, although thrombocytopenia has been reported in occasional patients with iron-deficiency anemia. Much less common is the development of thrombocytopenia following replenishment of iron stores.

**Case Presentation:**

We present the unusual case of a 39 year old African American female Jehovah's Witness who presented with a 10 month history of menorrhagia and pancytopenia. Laboratory investigations confirmed a severe iron deficiency. Since blood transfusion was unacceptable to her, she was started on intravenous iron replacement therapy. This precipitated a sudden drop in both her platelet and white blood cell counts. Histopathological examination of the bone marrow revealed a hypercellular marrow with orderly trilineage hematopoiesis, iron deficiency anemia, granulocytic hyperplasia, and mild megakaryocytic hypoplasia. Both her white blood cell and platelet counts recovered uneventfully with continuing iron supplementation. The possible mechanism for this phenomenon is discussed in this report.

**Conclusion:**

This case illustrates two rather uncommon associations of a very common problem. Severe iron deficiency anemia may be associated with pancytopenia and iron replacement may cause a transient decline in megakaryopoiesis and leukopoiesis. Severe iron deficiency should be added to the list of conditions leading to thrombocytopenia.

## Introduction

Iron deficiency anemia is the second most common nutritional deficiency in the United States [1] with an estimated 3.3 million women of child bearing age suffering from the condition [2].

Iron deficiency anemia is commonly associated with thrombocytosis with platelet counts between 500 to 700 × 10^9^/L [3]. The mechanism for this increase in platelet counts is thought to be the stimulation of platelet production by erythropoietin that is present in moderately increased levels in patients with iron-deficiency anemia [4]. Thrombocytopenia, although relatively uncommon in the setting of iron-deficiency anemia, has been reported before [5-7]. Another little known phenomenon is the development of thrombocytopenia following replenishment of iron stores [8,9]. A mild leukopenia has been reported with iron-deficiency anemia as well [10]. However, a decline in leukocyte counts following iron therapy has not been reported for leukocytes.

In this report, we present the unusual case of a severely iron deficient patient who presented initially with pancytopenia and then developed a precipitous decline followed by a recovery in platelet and leukocyte counts after iron replacement.

## Case presentation

A 39 year old African American female Jehovah's Witness with a ten-month history of menorrhagia was admitted for severe anemia and pancytopenia. Her hemoglobin on admission (Hgb) was 3.1 g/dL (normal range: 11.0 – 15.1 gm/dL), mean corpuscular volume was 58.6 fL (normal range: 79 – 97 fL) and red cell distribution width (RDW) was 35% (normal range: 11.3 – 15.5%). Reticulocyte count was low at 8000/mm^3 ^(normal range: 25 – 100 × 10^3^/mm^3^), and iron studies revealed a serum iron level of 17 μg/dL (normal range: 37 – 170 μg/dL), total iron binding capacity of 447 μg/dL (normal range: 250 – 450 μg/dL), percentage saturation of 4 and a ferritin level of <2 ng/mL (normal range: 10 – 100 ng/mL). Erythropoietin level was elevated at 9544 mU/mL (normal range: 0 – 27 mU/mL). White blood cell (WBC) count was 2.9 × 10^3^/μL (normal range: 4 – 11 × 10^3^/μL) with 46 segmented neutrophils, and her platelet count was 127 × 10^3^/mcL (normal range: 150 – 400 × 10^3^/μL), and coagulation parameters were normal.

Given her faith, transfusion was unacceptable, and iron supplementation was begun with intravenous iron sucrose complex given at a dose of 100 mg twice weekly. Two days after the initial iron infusion, her pancytopenia worsened with a drop in her platelets to 39 × 10^3^/mcL, hemoglobin decreased marginally to 2.7 gm/dL, and WBC count decreased to 1.6 × 10^3^/mcL. Given this sudden drop in hemoglobin, white blood cell counts and platelet counts, a bone marrow biopsy was performed to exclude a primary marrow disorder. Histopathological examination of the marrow revealed a hypercellular marrow, normal number of erythroid cells with a focus of left-shifted erythropoiesis, mildly decreased number of megakaryocytes. Evaluation of iron stores in the bone marrow showed decreased amounts of storage iron, but there was incorporation of iron. Ringed sideroblasts were not present in the bone marrow core sections.

**Figure 1:**
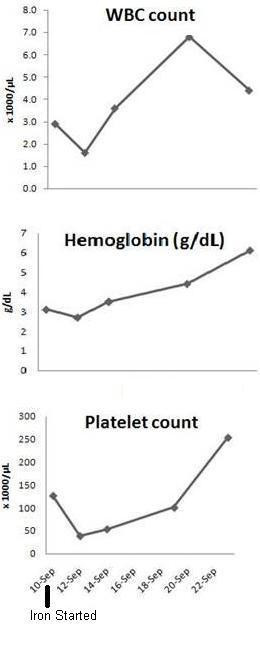
Temporal profile of hemoglobin, white blood cell and platelet counts.

After ten days of iron therapy, her WBC count had normalized to 5.9 × 10^3^/mcL, hemoglobin improved to 5.2 g/dL, and platelets were 195 × 10^3^/mcL. She received a total of 1500 mg of intravenous iron and at four months following completion of therapy, her hemoglobin (14.0 gm/dL), platelet counts (181 × 10^3^/mcL) and ferritin level (121 ng/mL) were within normal limits. Her WBC count was 3.8 × 10^3^/mcL, slightly below the lower limits of normal (Figure 1).

## Discussion

Our patient presented with pancytopenia. Given the extreme anemia and the marginal thrombocytopenia and leukopenia, she was initiated on iron replacement therapy in order to improve erythropoiesis. This led to a sudden decline in platelet and WBC counts (Figure 1).

Although iron deficiency is associated with a reactive thrombocytosis [3], increasing severity of the iron deficiency leads to normalization [11,12] and occasionally even decrease in platelet counts [5-7]. The exact mechanism of this is unclear but may be related to the alteration in the activity of iron dependent enzymes in thrombo- and leukopoiesis.

The mechanism of leukopenia in our patient may be related to the extremely high levels of erythropoietin seen in our patient. Animal experiments and in vitro studies using human hematopoietic stem cells have demonstrated that addition of erythropoietin to these stem cells down-regulates neutrophil production [13].

The sudden transient decrease in the platelet and leukocyte counts following iron therapy is probably related to the phenomenon of stem cell steal. Increased availability of the deficient erythrocyte precursor (iron) may have led to the diversion of the pluripotent hematopoietic stem cells towards erythropoiesis, at the expense of the other hematopoietic cell lines. In fact, the effect of erythropoietin therapy on platelet counts has been shown to be dependant on the adequacy of iron stores. When iron supply is inadequate, intense erythropoietin stimulation may cause thrombocytosis, but when iron supply is available, erythropoiesis predominates and megakaryopoiesis may be transiently decreased [14].

## Conclusion

This case illustrates two relatively uncommon findings in an extremely common disease. Iron deficiency anemia, if sufficiently severe, may be associated with decreased platelet and WBC counts and iron replacement therapy may cause a transient decline in megakaryopoiesis and leukopoiesis.

## Competing interests

The author(s) declare that they have no competing interests.

## Authors' contributions

AKG conceived the study, contributed to acquisition of data and drafted the initial manuscript and revised the manuscript prior to submission. NAS helped with the acquisition of the data, designing and drafting the initial draft, and revised the draft. WDH was the clinician responsible for making the treatment decisions on the patient. He also made contributions to acquisition of data, and revised the draft as necessary. All authors have read and approved the final manuscript.

## Consent

The patient was informed about the intent to publish this report and consented to the same in writing.
